# From barrier to enabler: Transforming language for global health collaboration

**DOI:** 10.1371/journal.pgph.0003237

**Published:** 2024-06-04

**Authors:** Marie Roseline Darnycka Bélizaire, Lynka Ineza, Ibrahima Socé Fall, Mitoha Ondo, Yap Boum

**Affiliations:** 1 Harvard LEAD Fellowship, Global Health Institute, Harvard School of Public Health, Boston, Massachusetts, United States of America; 2 School of Medicine, University of Alcala, Madrid, Spain; 3 World Health Organization, World Health Emergency Programme, Geneva, Switzerland; 4 Global Health Leadership Initiative, Yale School of Public Health, New Haven, Connecticut, United States of America; 5 World Health Organization, Global Neglected Tropical Disease Programme, Geneva, Switzerland; 6 Ministry of Health, Malabo, Equatorial Guinea; 7 Pasteur Institute of Bangui, Bangui, Central African Republic; 8 Homegrown Solution for Health, Yaoundé, Cameroon; 9 Faculty of Medicine and Biomedical Sciences, Department of Microbiology, University of Yaoundé I, Yaoundé, Cameroon; (McGill University, CANADA), and Catherine Kyobutungi (APHRC, KENYA)

## Why language matters

The biblical narrative of the Tower of Babel serves as a metaphor for the challenges and divisions created by the multiplicity and diversity of languages. According to the story, the construction of the Tower of Babel was seen as an act of defiance against divine will, leading to the creation of multiple languages, subsequently fragmenting humanity into groups unable to understand each other. This historical allegory underscores the importance of overcoming linguistic barriers to foster collaboration for the benefit of humankind. Linguists characterize language as a social determinant of health [1] that could be a barrier in healthcare leading to miscommunication between the healthcare professional and the patient. The latter may not have access to adequate healthcare services if they do not speak the language of the service providers, decreasing the quality of healthcare delivery and patient safety [2]. At the global level, non-English-speaking scientists have limited access to international conferences, significant funding, and scientific publications that are mainly in English [3].

**Fig 1:**
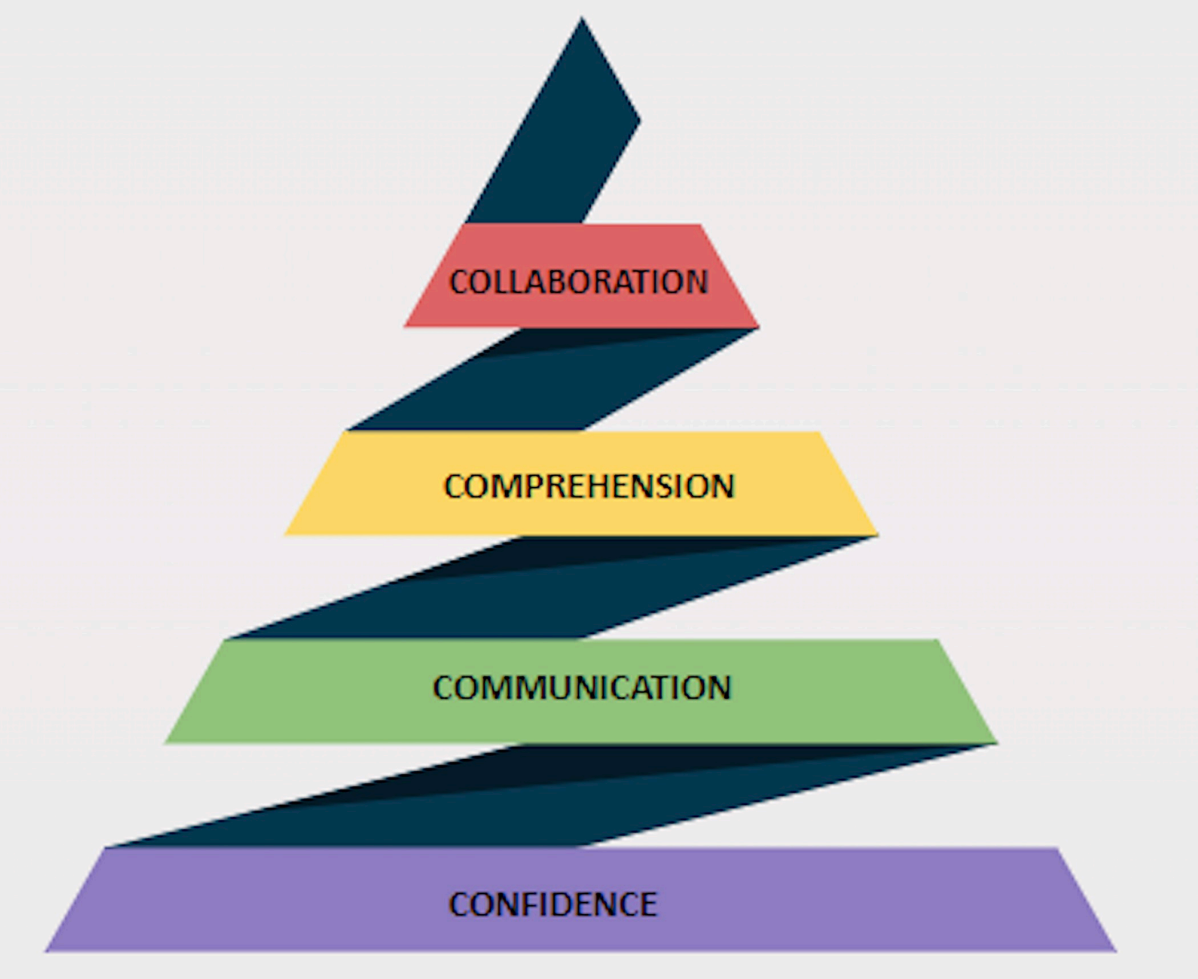
The foundation of global health success: The 4C pyramid of effective collaboration.

In the field of global health, effective collaboration is crucial for implementing inclusive projects that meet stakeholder goals and have a tangible impact on the communities they aim to enhance, ultimately improving quality of life. **C**ollaboration is at the top of the four C pyramid and is built upon a foundation of trust (**C**onfidence), **C**ommunication, understanding (**C**omprehension), and the collective pursuit of a common goal (Fig 1). These elements are critical for successful partnerships. Unfortunately, language disparities can hinder these foundational layers, impacting trust, limiting communication, obstructing understanding, and blocking collaboration.

## Linguistic obstacles to achieving global health equity

The impact of language limitations is evident in the global health research community. Francophone, Spanish and Lusophone researchers often encounter obstacles in accessing training or funding opportunities predominantly available in English [3]. Despite the ubiquity of outbreaks, resources and opportunities remain disproportionately available to English-speaking communities [4]. The recent Marburg outbreak in Equatorial Guinea, a Spanish and Portuguese-speaking country, exemplified the challenges faced by international public health experts predominantly fluent in French or English. The rarity of experts proficient in Portuguese, Spanish, and English underscores the need for a more linguistically diverse global health workforce [5].

Language barriers can also impact the visibility of non-English researchers. In a systematic review assessing the contribution of researchers in Africa to research on HIV, tuberculosis, malaria, Buruli ulcer, Ebola, and salmonellosis, it was found that the first and last authors were predominately drawn from Anglophone countries. There is a geographical inequity in representing African first and last authors involved in highly and moderately researched infectious disease research. Anglophone countries like South Africa, Ethiopia, Nigeria, Kenya, and Ghana are the most represented countries of origin of African first and last authors [6].

Moreover, an analysis of international research funding reveals a significant disparity, with a predominant share originating from English-speaking countries and disproportionately benefiting Anglophone African countries over their Francophone counterparts. In the US, more than 90% of NIH funding in 2022 to Africa is toward English-speaking countries, limiting the opportunities for other countries to contribute to research in global health [7]. This imbalance underscores language as both a barrier and a catalyst for inequitable partnerships and their impact.

## Effective strategies for bridging language gaps

Efforts are underway to address these challenges. For example, the Yale Global Health Leadership Initiative (GHLI) is addressing inequities in the design and implementation of leadership and management programs in Africa [8]. GHLI is implementing equitable training programs in both Anglophone and Francophone countries through the engagement of regional partners in the adaptation and contextualization of the programs. Some of the programs implemented in French-speaking countries include: (1) The Expanded Program on Immunization Leadership and Management Program (EPI LAMP); (2) The Strategic Training Executive program for supply chain managers; and (3) the Leadership and mentorship program. For example, EPI LAMP reached 27 countries with a total of 100 participants across 5 cohorts that includes 40 participants representing 12 French-speaking countries. After assessing the change in leadership and management competencies in the first 3 cohorts, there was no difference observed in the results between the French and English speaking countries which highlights the need for equal access to such programs in French-speaking countries [8, 9].

Similarly, The World Health Organization (WHO) launched the first online learning course on COVID-19 in January 2020 for frontline workers and decision-makers using WHO’s emerging evidence-based knowledge for managing the pandemic. The course “Introduction to COVID-19” was hosted on the WHO Health Emergencies learning platform OpenWHO.org. The platform hosts courses in 55 different languages with 10.4 million words translated [10, 11].

Major scientific journals are making strides toward inclusivity by publishing articles in the authors’ native languages as supplementary material. This practice, adopted by journals such as *Nature Behavior*, *The Lancet*, and the *New England Journal of Medicine*, enhances accessibility and ownership of research findings among communities most in need [12–14]. However, the ultimate goal of ensuring that research beneficiaries can fully comprehend and utilize these findings remains a work in progress.

## Artificial intelligence: A catalyst for achieving global health equity

Technology has improved access to translation. With the acceleration of artificial intelligence (AI) large language models, we can further improve interpreter access through real-time, automated translation. To improve the scientific writing of non-native English speakers, Artificial intelligence-based programs can correct grammatical errors and improve writing style [15, 16].

Similarly, using artificial intelligence, connecting platforms such as The Village (https://globalhealthvillage.org/) make fruitful matches in resources, ideas, and collaboration. It moves beyond the limitations, including language, by bringing together those who have resources and those who have needs, thus generating demonstrable products of mentorship, innovation, and programs. Nonetheless, the deployment of AI must be guided by carefully crafted policies and guidelines that prevent AI-generated, fake papers, as well as safeguard confidentiality and promote ethical practices.

## Leveraging language as a key enabler for global health equity: A path forward

The proposal for greater linguistic inclusivity in scientific publishing and discourse has challenges. Due to language barriers, scientists often face difficulties communicating with partners and populations during studies and public health responses. Technology and artificial intelligence offer solutions to bridge these gaps, enabling translation and facilitating communication across language barriers.

To further reduce language barriers, promote equity, and improve language inclusion within the global health community, the following five recommendations are proposed:

1. Scientific journals should offer access to translations of published articles in the language of the study’s location to ensure local accessibility, proper use, and relevance.
2. Conference and meeting organizers should provide translation services and allow scientists to present and discuss in their official languages.
3. Every institution involved in global health should leverage funding to train researchers and public health experts in the language spoken in the country where they will operate to build capacity among international stakeholders and to enhance effectiveness and cultural and scientific exchange.
4. Funders should allow for grant applications and grant reports to be submitted in the local language.
5. In the meantime, scientists and public health experts should make an effort to learn English which is the current lingua franca.

These recommendations aim to foster a more inclusive, equitable, and effective global health community by bridging linguistic divides and promoting cultural understanding. Therefore, language should not merely be a communication medium but a facilitator for effective collaboration among global health scientists.

## References

[1] Showstack Rachel, Santos Maricel G., Feuerherm Emily, Jacobson Holly & GM. Language as a social determinant of health: an applied linguistics perspective on health equity [Internet]. American Association for Applied linguistic. 2019 [cited 2024 Feb 21]. Available from: https://www.aaal.org/news/language-as-a-social-determinant-of-health-an-applied-linguistics-perspective-on-health-equity.

[2] Al Shamsi H, Almutairi AG, Al Mashrafi S, Al Kalbani T. Implications of language barriers for healthcare: A systematic review. Oman Med J. 2020;35(2):1–7. doi: 10.5001/omj.2020.40 32411417 PMC7201401

[3] Amano T, Ramı´rez-Castañeda V, Berdejo- Espinola V, Borokini I, Chowdhury S GM. Peer Review: The cost of being a non-native English speaker in science. PLoS Biol [Internet]. 2023;21(7):1–27. Available from: https://journals.plos.org/plosbiology/article?id=10.1371/journal.pbio.3002184. 10.1371/journal.pbio.3002184 PMC10353817 37463136

[4] Ransing R, Vadivel R, Halabi S El, Jatchavala C, Shalbafan M, Noël C, et al. Language as Multi-Level Barrier in Health Research and the Way Forward. Indian J Psychol Med. 2023;45(1):65–8. doi: 10.1177/02537176211052071 36778626 PMC9896124

[5] Sibomana O, Kubwimana E. First-ever Marburg virus disease outbreak in Equatorial Guinea and Tanzania: An imminent crisis in West and East Africa. Immunity, Inflamm Dis. 2023;11(8):1–10. doi: 10.1002/iid3.980 37647447 PMC10461415

[6] Mbaye R, Gebeyehu R, Hossmann S, Mbarga N, Bih-Neh E, Eteki L, et al. Who is telling the story? A systematic review of authorship for infectious disease research conducted in Africa, 1980–2016. BMJ Glob Heal. 2019;4(5). 10.1136/bmjgh-2019-001855 PMC6830283 31750001

[7] Jessica E. Haberer M.D., Yap Boum II P. Behind-the-Scenes Investment for Equity in Global Health Research. N Engl J Med. 2023;387–90. 10.1056/NEJMp2213809 36724379

[8] Ineza L, Bechtold K, Mwisongo A, Kwedi Nolna S, Linnander EL. Building leadership and management competencies of national immunization teams in 16 Gavi-eligible countries through the EPI leadership and management programme. Vaccine [Internet]. 2022;40(26):3581–7. Available from: doi: 10.1016/j.vaccine.2022.04.070 35581100

[9] Linnander E, Nolna SK, Mwinsongo A, Bechtold K, Boum Y. Reaching across the linguistic divide in management and leadership education. Lancet Glob Heal [Internet]. 2019;7(9):e1177. Available from: doi: 10.1016/S2214-109X(19)30256-6 31402002

[10] Utunen H, Van Kerkhove MD, Tokar A, O’Connell G, Gamhewage GM, Fall IS. One year of pandemic learning response: Benefits of massive online delivery of the world health organization’s technical guidance. JMIR Public Heal Surveill. 2021;7(4). doi: 10.2196/28945 33881404 PMC8061891

[11] Utunen H, Ndiaye N, Attias M, Mattar L, Tokar A, Gamhewage G. Multilingual Approach to COVID-19 Online Learning Response on OpenWHO.org. Stud Health Technol Inform. 2022;289(2019):192–5. doi: 10.3233/SHTI210892 35062125

[12] Ngou O, Nzoumbou-Boko R, Bakamba P, Boum Y. R21/Matrix-M malaria vaccine: a vital tool in the arsenal against malaria, not a silver bullet. Lancet Infect Dis [Internet]. 2024;3099(23):23–4. Available from: 10.1016/S1473-3099(24)00010-0. 38342103

[13] Roca A, Boum Y, Wachsmuth I. A francophone call for more inclusive global health research. Lancet Glob Heal. 2019;7(6):e701–2. 10.1016/S2214-109X(19)30175-5 31000374

[14] Ser-E. Scientific publishing has a language problem. Nat Hum Behav. 2023;7(7):1019–20. 37488202 10.1038/s41562-023-01679-6

[15] Bakdash L, Abid A, Gourisankar A, Henry TL. Chatting Beyond ChatGPT: Advancing Equity Through AI-Driven Language Interpretation. J Gen Intern Med [Internet]. 2023;(0123456789):492–5. Available from: doi: 10.1007/s11606-023-08497-6 37904073 PMC10897100

[16] Giglio A Del, da Costa MUP. The use of artificial intelligence to improve the scientific writing of non-native english speakers. Rev Assoc Med Bras. 2023;69(9):1–5. doi: 10.1590/1806-9282.20230560 37729376 PMC10508892

